# The effect of hypoxic postconditioning after severe hypobaric hypoxia on the expression of Cu, Zn-SOD in hippocampus and neocortex of rats

**DOI:** 10.1186/2193-1801-4-S1-P45

**Published:** 2015-06-12

**Authors:** Sergei Alexandrovich Stroev, Tatiana Sergeevna Glushchenko, Ekaterina Iosifovna Tjul’kova, Mikhail Olegovich Samoilov

**Affiliations:** School of Medicine, University of Tampere, Finland; I.P. Pavlov Institute of Physiology of RAS, Russian Federation, Russia

**Keywords:** Postconditioning, hypoxia, Cu, Zn-superoxide dismutase

Oxidative stress is a key mechanism of cellular damage during and after severe hypoxia. Accordingly, up-regulation of expression and activity of endogenous antioxidants is an important mechanism of cellular adaptation to hypoxia. Do endogenous antioxidants take part in postconditioning-induced neuroprotective mechanisms similarly to their participation in preconditioning-induced ones? In the present work the effect of postconditioning by 3-trial mild hypobaric hypoxia (360 Torr, 2 h, once a day) after 1 session of severe acute hypobaric hypoxia (180 Torr, 3 h) on the expression of Cu, Zn-superoxide dismutase (Cu, Zn-SOD) was studied by immunocytochemical analysis in areas CA1, CA2, CA3, CA4 and DG of hippocampus and in frontoparietal neocortex (NC) of male Wistar rats. Two time points were examined: 3 h after the last session of postconditioning that was 3 days after severe hypoxia and 24 h after the last session of postconditioning that was 4 days after severe hypoxia. It has been shown that postconditioning significantly increases the total number of Cu, Zn-SOD-immunoreactive cells (Nt) at least in two areas of hippocampus studied (CA2 and DG) compared to non-postconditioned rats at 3 days but not at 4 days after severe hypoxia. In contrast, in NC of postconditioned rats, Nt tends to increase compared to non-postconditioned animals at 4 days but not at 3 days after severe hypoxia. The effect of postconditioning on the number of intensely expressing Cu, Zn-SOD neurons differs in various areas and at various time points. The modification of Cu, Zn-SOD expression in some areas of hippocampus and NC, induced by 3-trial hypoxic postconditioning, correlates with the prevention of massive delayed apoptotic neuronal death and amelioration of functional disorders caused by severe hypoxia. Thus, Cu, Zn-SOD and other endogenous antioxidants may play, apparently, an important role in the treatment of severe hypoxia/ischemia stroke by postconditioning in brain neurons.

